# Prof. C. A. Lee

**Published:** 1850-01

**Authors:** 


*Prof. C. A. Lee.*—The friends of Dr. Lee will be gratified to be informed of his safe return from Europe, with improved health. His course of instruction in Pathology and Materia Medica, in the University of Buffalo, will commence with the commencement of the present month.

